# HIV Progression Perturbs the Balance of the Cell-Mediated and Anti-Inflammatory Adaptive and Innate Mycobacterial Immune Response

**DOI:** 10.1155/2016/1478340

**Published:** 2016-03-02

**Authors:** Andrew R. DiNardo, Anna M. Mandalakas, Gugu Maphalala, Godwin Mtetwa, Temhlanga Mndzebele, Piluca Ustero, Makhosazana Hlatshwayo, Emily M. Mace, Jordan S. Orange, George Makedonas

**Affiliations:** ^1^The Global Tuberculosis Program, Texas Children's Hospital, Immigrant and Global Health, Department of Pediatrics, Baylor College of Medicine, Houston, TX 77030, USA; ^2^National Tuberculosis Reference Laboratory, Swaziland Health Laboratory Services, Ministry of Health, Mbabane, Swaziland; ^3^The Global Tuberculosis Program, Texas Children's Hospital, Baylor College of Medicine Children's Foundation-Swaziland, Mbabane, Swaziland; ^4^Department of Pathology and Immunology, Baylor College of Medicine, Texas Children's Hospital Center for Human Immunobiology, Department of Pediatrics, Houston, TX 77030, USA

## Abstract

*Introduction*. Our objective is to understand how HIV infection increases the risk of progression from latent tuberculosis (TB) to active disease. We understand now that immunity is a balance of competing immune responses by multiple cell types. Since T-lymphocyte production of interferon-gamma (IFN-*γ*) in response to* Mycobacterium tuberculosis (Mtb*) antigens fails to differentiate disease from latent infection, we applied a comprehensive profiling methodology to define immune biomarkers that reliably predict a patient's TB risk.* Methods*. We established a cohort of HIV-infected adults with TB disease from Swaziland. Multiparametric flow cytometry was used to quantify the mycobacterial-specific anti-inflammatory (IL-4 and IL-10) and proinflammatory (IFN-*γ*) immune response.* Results*. From 12 HIV-infected Swaziland patients with TB disease, the CD4^+^, CD8^+^, Double Negative, and CD56^+^CD3^−^ lymphocytes increase their IL-4 : IFN-*γ* ratio as HIV disease worsens (Spearman *r* of −0.59; −0.59; −0.60; and −0.59, resp.; *p* < 0.05). Similarly, HIV severity is associated with an increased IL-10 : IFN-*γ* ratio (Spearman *r* of −0.76; *p* = 0.01).* Conclusion*. As HIV disease progresses, both the adaptive and innate branches skew away from an inflammatory and towards anti-inflammatory phenotype.

## 1. Introduction

Compared to HIV-uninfected individuals living with* Mycobacterium tuberculosis* infection (MTBI), people living with HIV (PLHIV) possess a 20–37-fold higher risk of developing tuberculosis (TB). This disease accounts for approximately 25% of all HIV-related deaths [1–3]. The prevailing paradigm explaining the immunologic mechanism underlying this epidemiologic phenomenon is that HIV eliminates CD4 T cells, including those presumed to be essential for* Mtb* control. Interferon-gamma (IFN-*γ*) production by T cells in response to an* Mtb* stimulus is the standard immunologic test for MTBI; however, this measurement does not differentiate MTBI from TB disease. Since progression along the TB disease spectrum is a major predictor of mortality for PLHIV, there is a critical need for clinical immune biomarkers that reliably predict a patient's precise TB condition.

Multiparametric analyses of the immune system have illuminated our insight of the mechanisms of disease pathogenesis and protection [4–10]. It is clear now that the immune response to any given pathogen is complex and multifaceted [11]. Thus, relying on a single immunologic measure to yield an accurate diagnosis is suboptimal and perhaps even misleading. Understanding the dynamics of an immune equilibrium between competing responses may improve our predictive capacity for clinical outcome (see Supplemental Figure 1 in Supplementary Material available online at http://dx.doi.org/10.1155/2016/1478340). For example, during TB infection, anti-inflammatory agents (such as IL-4, IL-10, and immune checkpoint inhibitors) prevent host tissue destruction due to excessive cell-mediated immunity [11–14]. In contrast, excessive IL-4 or IL-10 production may result in a decreased containment of intracellular* Mtb* and therefore theoretically increased risk of TB disease progression [15, 16].

To evaluate if the balance between pro- and anti-inflammatory CD4 T cell responses from PLHIV subjects with TB correlates with TB progression, we quantified the production of mycobacteria-specific IFN-*γ*, IL-4, and IL-10 simultaneously. We hypothesized a direct relationship between HIV disease severity and a skewing of the immune equilibrium away from cell-mediated and towards an anti-inflammatory profile. In addition, we profiled the cytokine responses from CD8, CD56^+^CD3^−^ NK cells, and CD3^+^CD4^−^CD8^−^CD56^−^ (Double Negative T Lymphocytes). As the CD4 T cell compartment wanes with advancing HIV disease status, these immune cell types gain prominence. For PLHIV at risk for TB disease development, our objective is to elucidate the effect of HIV progression on the balance of the antimycobacterial immune response.

## 2. Methods

Adults with microbiologically confirmed (GeneXpert Mtb/RIF or culture positive) pulmonary TB (*N* = 12) were enrolled after completing written informed consent. Ethical approval was obtained by all relevant bodies including Baylor College of Medicine, Swaziland Children's Foundation, Baylor College of Medicine IRB, and the Swaziland Ethics Committee. Blood collected into ACD tubes was transported to the laboratory, within 4 hours, and PBMCs were isolated using Ficoll density gradient as previously described [6]. To elicit an immune response by the adaptive and innate immune responses, PBMCs were stimulated with* Mycobacterium bovis* whole cell lysate (5 *μ*g/mL) for 16 hours which was obtained through BEI Resources, NAID, NIH (*Mycobacterium bovis*, Strain AF 2122/97 ATCC® BAA-935*™*, Whole Cell Lysate, NR-31211). DMSO, the solvent that antigen reconstituted, was the negative control. Staphylococcal enterotoxin B (SEB; Millipore) was used as the positive controls. Stimulation occurred in the presence of Brefeldin A (Sigma, 1 *μ*g/mL), Golgi-Stop (BD Biosciences, 0.7 *μ*g/mL), and costimulatory antibodies CD28/CD49 (BD Biosciences, 2.5 *μ*g/mL). Following stimulation, cells were washed and stained for viability (Ghost dye, Tonbo Biosciences), surface antigens, CD3 (BioLegend), CD8 (BioLegend), CD4 (eBioscience), CD14 (BioLegend), and CD56 (BioLegend), and intracellular proteins, IFN-*γ* (Life Technologies), IL-4 (BD Bioscience), and IL-10 (BD Bioscience). The cells were acquired on a BD LSR II Fortessa Flow Cytometer as previously described [6]. Representative gating to define the functional responses is illustrated in Supplementary Figure 1. Flow cytometry and statistical analyses were performed using FlowJo X (TreeStar) and GraphPad 6.0 (GraphPad Software), respectively. Correlation of nonparametric data was performed using Spearman's test.

## 3. Results

**Figure 1:**
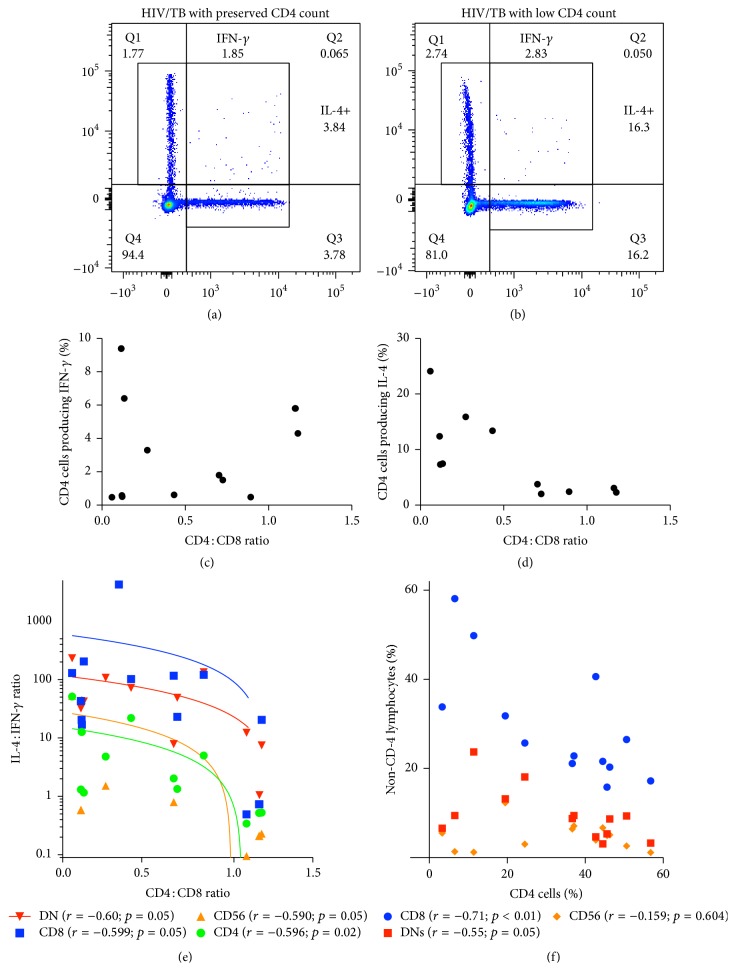
HIV progression increases immune skewing towards IL-4 and away from IFN-*γ* amongst CD4^+^, CD8^+^, Double Negative (CD3^+^CD4^−^CD8^−^), and CD56^+^ lymphocytes. PBMCs from HIV-TB coinfected patients were stimulated with* Mycobacterium bovis (M. bovis*) lysate to measure the CD4, CD8, NK (CD56^+^CD3^−^), and Double Negative (CD3^+^CD4^−^CD8^−^) lymphocyte response. (a and b) Representative dot plots illustrating definition of* M. bovis* IFN-*γ* and IL-4 responses based on CD4 cell count. Full gating strategy is available in Supplemental Figure 2. (c) Percentage of CD4 cells producing mycobacterial-specific IFN-*γ* based on CD4 : CD8 ratio. (d) Percentage of CD4 cells producing mycobacterial-specific IL-4 based on CD4 : CD8 ratio. (e) Spearman correlation of the mycobacteria-specific IL-4 : IFN-*γ* ratio based on the CD4 : CD8 ratio for CD4 cells, CD8 cells, CD56 cells, and Double Negative Lymphocytes. (f) Percentage of CD8 lymphocytes, CD56 lymphocytes, or Double Negative Lymphocytes based on declining CD4 cell percentage.

While CD4 T cell IFN-*γ* production remains the dogmatic measurement of mycobacterial immunity, it fails to predict which individuals are at increased risk of disease progression [17]. To this end, we hypothesized a more accurate means via a comprehensive immune profile balancing the anti-inflammatory cytokines (IL-4 and IL-10) compared to inflammatory ones (IFN-*γ*). We measured the mycobacteria-specific IL-4 : IFN-*γ* and IL-10 : IFN-*γ* ratios from CD4, CD8, CD56, and Double Negative (CD3^+^CD4^−^CD8^−^CD56^−^) cells. Representative dot plots are available in Figures 1(a) and 1(b) and the gating strategy is available in Supplemental Figure 2. As a proxy for HIV progression, we calculated the CD4 : CD8 ratio.

Participants had a mean age of 24 (range 16–38), 58% female with 100% on Antiretroviral Therapy (ART) (see Table 1). As HIV progresses and the percentage of CD4 cells decreases, the percentage of lymphocytes that are CD8^+^ or DN increases (Figure 1(f)); this association was not seen with CD56^+^CD3^−^ NK cells. Similar to previous descriptions [18], the percentage of CD4 T cells producing mycobacteria-specific IFN-*γ* did not correlate with CD4 : CD8 ratio (Spearman *r* = 0.059; *p* = 0.83) (Figure 1(c)), while the percentage of IL-4 producing CD4 cells increased with HIV progression (Figure 1(d)). When considering the balance, IL-4 : IFN-*γ* increased as the CD4 : CD8 ratio decreased (Spearman *r* of −0.596; *p* = 0.02; Figure 1(e)). The correlation of increasing IL-4 : IFN-*γ* ratio with decreasing CD4 : CD8 ratio occurred in both the adaptive (CD4 and CD8) and innate (CD56^+^CD3^−^ NK cell) response as well as in the immune response of Double Negative (DN) Lymphocytes (Figure 1(e)).

**Figure 2:**
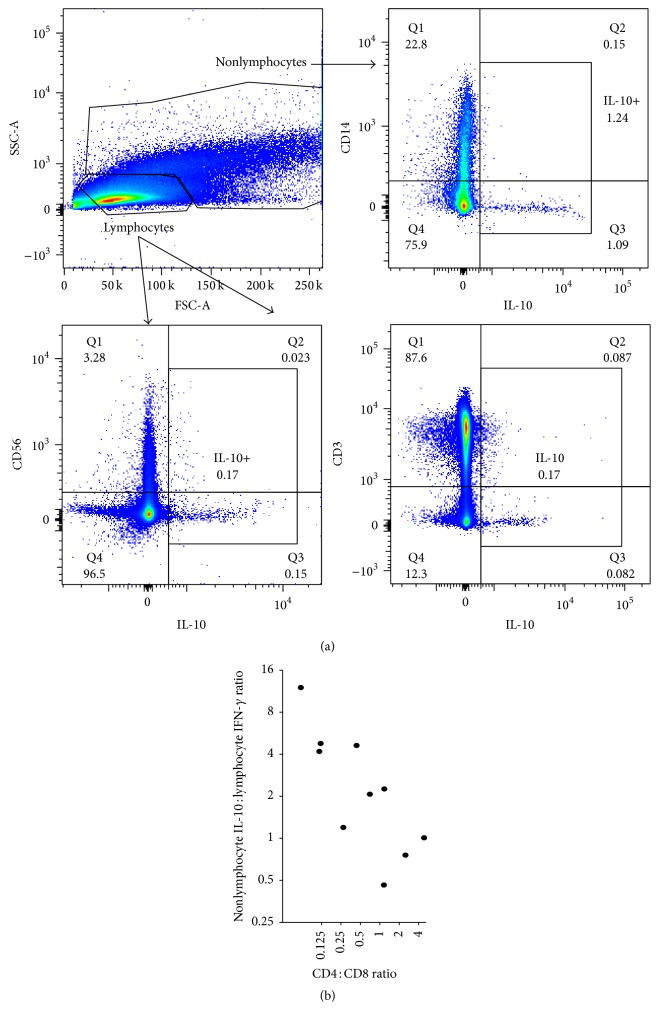
HIV progression increases the immune skewing towards nonlymphocyte IL-10 and away from lymphocyte IFN-*γ*. PBMCs from HIV-TB coinfected patients were stimulated with* Mycobacterium bovis (M. bovis*) lysate to evaluate the etiology of IL-10 production. (a) Representative dot plots showing the relative contribution of cell types towards IL-10 production. Side scatter (SSA-A) and forward scatter (FSA-A), CD3, CD56, and CD14 were used to evaluate the lymphocyte or nonlymphocyte contribution of IL-10 production. (b) Spearman correlation of the mycobacteria-specific nonlymphocyte IL-10 : total lymphocyte IFN-*γ* production based on the CD4 : CD8 decline.

While there are many cells capable of producing IL-10 (lymphocytes, granulocytes, monocytes, macrophages, and dendritic cells) [19], their production occurred predominantly in nonlymphocytes (Figure 2(a)). In contrast, the contribution from CD56^+^ NK or CD3^+^ T cells was minimal (Figure 2(a)). Therefore, we correlated the IL-10 production from nonlymphocytes to the total percentage of lymphocytes producing mycobacteria-specific IFN-*γ* as an appropriate measure for the balance of pro- and anti-inflammatory immune responses (Figure 2(a)). As HIV disease progressed (as measured by declining CD4 : CD8 ratio), the nonlymphocyte IL-10 : lymphocyte IFN-*γ* ratio increased (Spearman *r* = −0.76; *p* = 0.01) (Figure 2(b)). Therefore, while the percentage of CD4 T cells producing IFN-*γ* did not correlate with HIV progression, the balance of anti-inflammatory (IL-4 or IL-10) and cell-mediated immune responses did.

## 4. Discussion 

Combatting the HIV-TB epidemics ravaging vulnerable populations is going to require significant investments in clinical care and also cutting edge basic and translational research to discover new diagnostics, identify pathways for immunotherapeutics, and develop effective vaccine platforms. Here, amongst a cohort of Swaziland PLHIV with TB disease, we show that the benefit of applying a comprehensive and balanced profile of antimycobacterial immunity is of paramount importance. For progression to TB disease, HIV infection is the greatest risk factor [1–3] and, therefore, identification of immune correlates of TB progression amongst PLHIV is of utmost importance. The central doctrine of mycobacterial immunity relies on CD4^+^ T cell IFN-*γ* production; however, this measurement fails to differentiate infection from disease, predict those at high risk for progression, or predict vaccine immunogenicity [17, 18]. Thus, there is an urgent need for reliable immune biomarkers of protection, as well as disease advancement with recent research delineating the roles of cytotoxic T lymphocytes (CD8^+^) [4, 20], NK cells [18], Double Negative cells, monocytes, and macrophages [18].

In contrast to the classic approach of only evaluating the cell-mediated mycobacterial immune response, we demonstrate how the simultaneous evaluation of cell-mediated and anti-inflammatory responses improves our understanding of the immune processes at play. In contrast to the premise that IFN-*γ* defines mycobacterial immunity best, we show that as HIV disease progresses, both adaptive and innate lymphocyte lineages polarize towards an IL-4-dominated anti-inflammatory response. The evidence for HIV promoting a Th2 skewing of the immune response is controversial as it is dependent on the methodology (PCR [21], ELISA [22, 23], or flow cytometry [24]) and type of stimuli (antigen-specific versus PMAI/I). Here, using multiparametric flow cytometry to measure the antigen-specific response, we show that HIV progression causes an increase in the CD4 T cell IL-4: IFN-*γ* ratio. For the first time to our knowledge, we demonstrate a negative correlation between IL-4 and IFN-*γ* that occurs amongst not only CD4^+^ T cells, but also CD8^+^ T cells, Double Negative Lymphocytes, and NK cells.

These preliminary findings raise interesting questions about the pathophysiology of TB disease progression in the HIV setting. One possible explanation for the increased IL-4 : IFN-*γ* ratio may be increased cell turnover. GATA-3, the master transcription factor promoting IL-4 and inhibiting IFN-*γ*, is required for thymic development [25]. It is plausible that as HIV progression results in high lymphocyte turnover, the increasing percentage of GATA-3^+^ naïve immune cells explains the etiology of IL-4 skewing. If this were the explanation, one would expect the ratio to normalize the longer an individual is on suppressive ART.

Our data suggest that CD3^+^ T cells do not produce significant pathogen-specific IL-10. In contrast, the ratio of nonlymphocyte IL-10 : lymphocyte IFN-*γ* correlates inversely with HIV progression. Animal models [12, 14, 26, 27] suggest that an appropriate balance of anti-inflammatory to proinflammatory immunity is needed to control intracellular* Mtb*. Correlation of these animal models with longitudinal human data is needed to identify the ratio at which IL-10 is protective versus when it becomes detrimental to the protective cell-mediated response.

These preliminary findings in this communication are significant insofar as they begin to define the relationship between HIV disease severity and the immune response equilibrium amongst multiple lymphocyte lineages. Replication of these primary findings in longitudinal cohorts of TB exposed PLHIV promises to assess the predictive utility IL-4 : IFN-*γ* or IL-10 : IFN-*γ* ratios, further elucidating the mechanisms of* Mtb* protective immunity and therefore supporting development of targeted immune therapies.

## Supplementary Material

Supplemental 1: Proposed graphic abstract for evaluating the balance of pro-inflammatory, humoral immunity, wound healing and anti-inflammatory roles of lymphocytes.
Supplemental 2: Gating strategy for evaluating Lymphocytes. Single cells are first gated upon, followed by lymphocytes based on cell size (SSC vs FSC scatter and then live cells. Cell lineage undergoes negative selection followed by a final positive selection and then functional gating is performed.

## Figures and Tables

**Table 1:** 

Age	Sex	CD4 count	CD4 : CD8 ratio
20	F	92	0.12
36	M	70	0.2
17	F	40	0.11
18	M	825	1.35
23	F	325	0.3
16	F	132	0.06
21	M	331	0.15
21	M	86	0.69
23	F	668	0.63
22	F	640	0.62
31	F	369	0.89
52	M	785	1.23
