# Supplementary figures and images for: HIV Progression Perturbs the Balance of the Cell-Mediated and Anti-Inflammatory Adaptive and Innate Mycobacterial Immune Response
Source: Mediators Inflamm. 2016 Mar 2;2016:1478340. doi: 10.1155/2016/1478340 (PMC4781991; doi:10.1155/2016/1478340)

Supplemental Figure 1

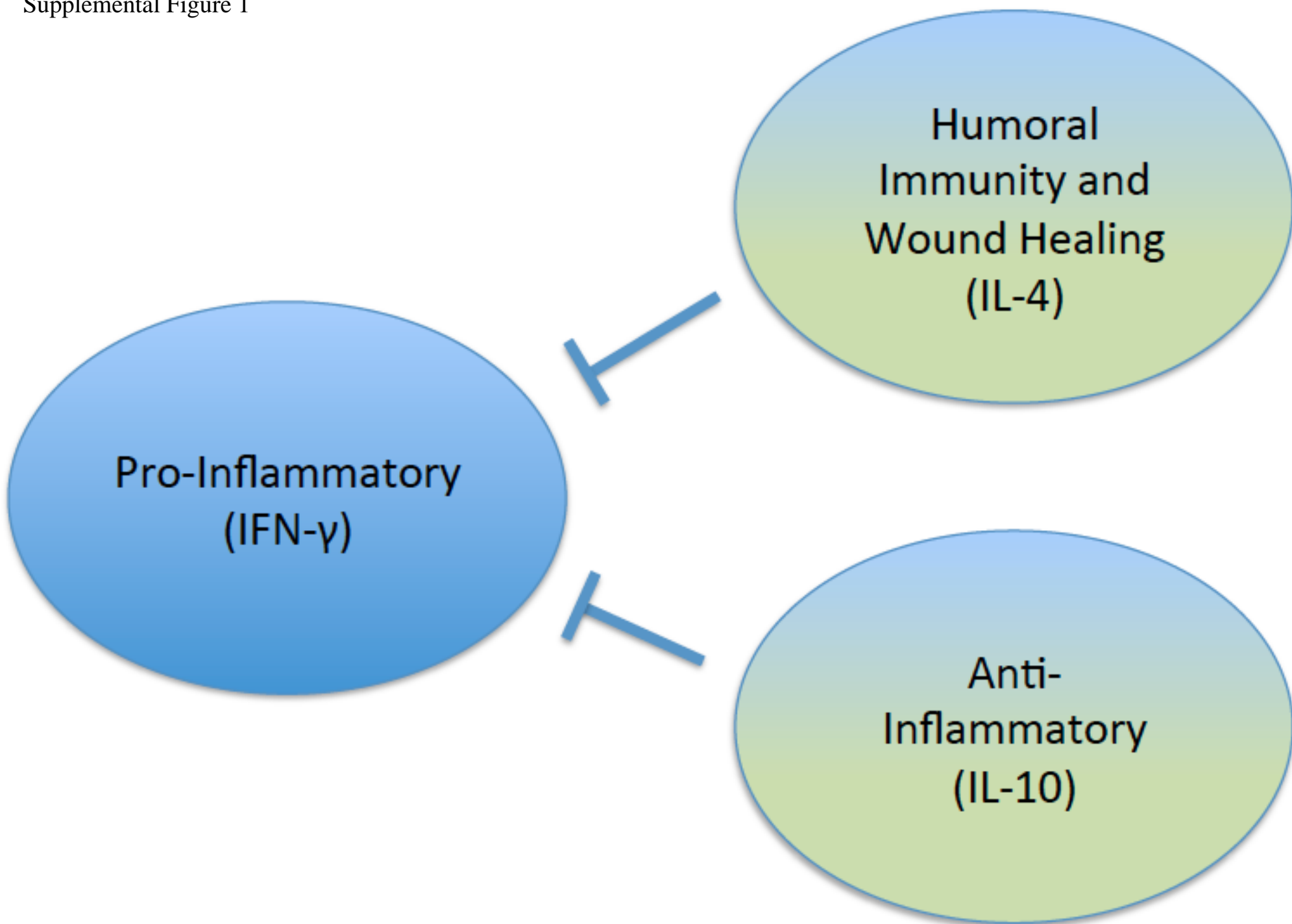

Supplemental Figure 2

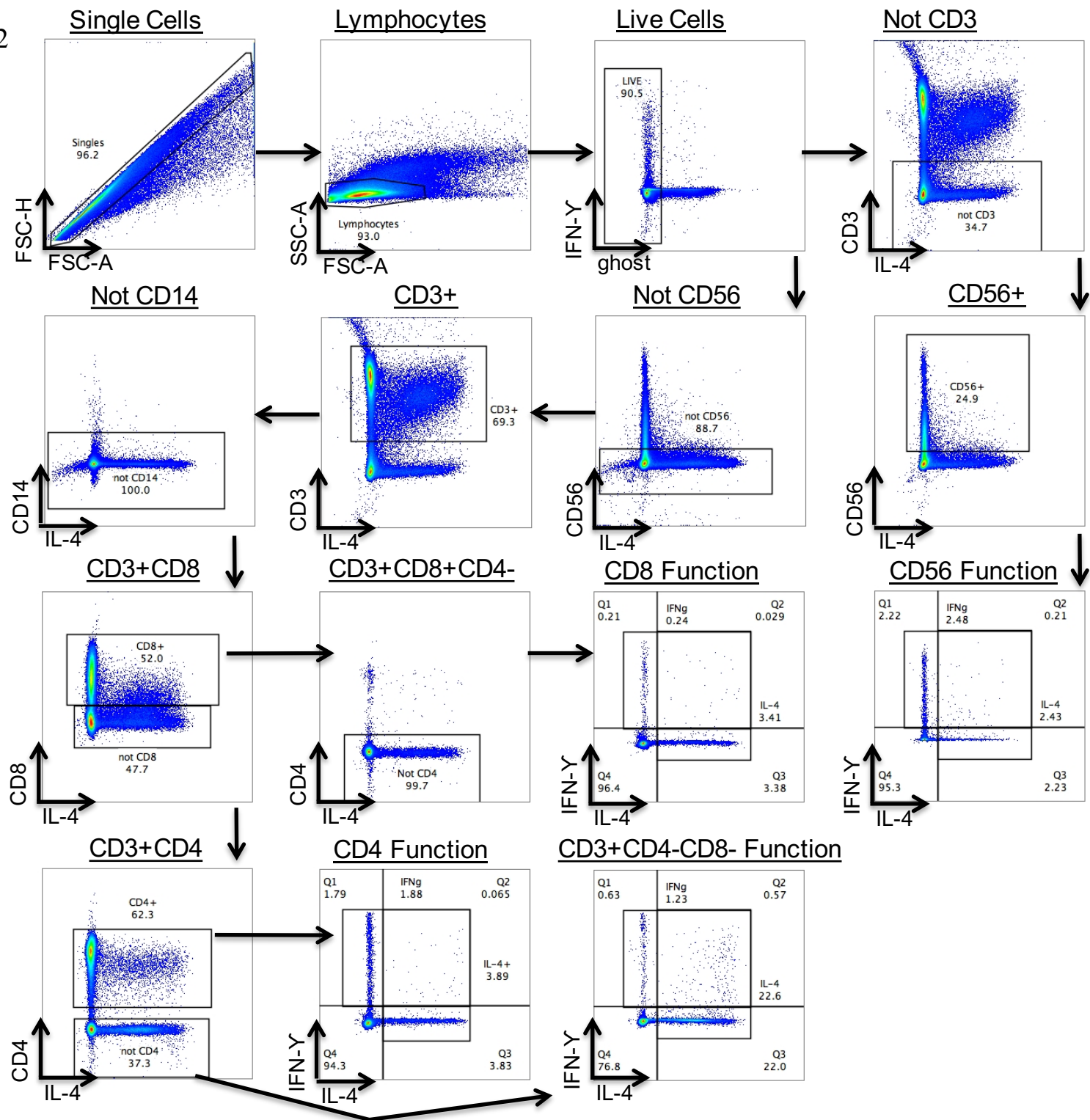

Supplement: Supplementary file 1 — Supplemental 1: Proposed graphic abstract for evaluating the balance of pro-inflammatory, humoral immunity, wound healing and anti-inflammatory roles of lymphocytes. Supplemental 2: Gating strategy for evaluating Lymphocytes. Single cells are first gated upon, followed by lymphocytes based on cell size (SSC vs FSC scatter and then live cells. Cell lineage undergoes negative selection followed by a final positive selection and then functional gating is performed. [file 1478340.f1.pdf]
